# Phrenic nerve stimulation prevents diaphragm atrophy in patients with respiratory failure on mechanical ventilation

**DOI:** 10.1186/s12890-021-01677-2

**Published:** 2021-10-08

**Authors:** Michal Soták, Karel Roubík, Tomáš Henlín, Tomáš Tyll

**Affiliations:** 1 Department of Anesthesiology and Intensive Care, First Faculty of Medicine, Charles University and Military University Hospital, U Vojenské nemocnice 1200, Prague, 169 02 Czech Republic; 2 Department of Biomedical Technology, Faculty of Biomedical Engineering, Czech Technical University, Prague, Czech Republic

**Keywords:** Weaning from mechanical ventilation, Diaphragm atrophy, Phrenic nerve, Ultrasound

## Abstract

**Background:**

Diaphragm atrophy and dysfunction is a major problem among critically ill patients on mechanical ventilation. Ventilator-induced diaphragmatic dysfunction is thought to play a major role, resulting in a failure of weaning. Stimulation of the phrenic nerves and resulting diaphragm contraction could potentially prevent or treat this atrophy. The subject of this study is to determine the effectiveness of diaphragm stimulation in preventing atrophy by measuring changes in its thickness.

**Methods:**

A total of 12 patients in the intervention group and 10 patients in the control group were enrolled. Diaphragm thickness was measured by ultrasound in both groups at the beginning of study enrollment (hour 0), after 24 hours, and at study completion (hour 48). The obtained data were then statistically analyzed and both groups were compared.

**Results:**

The results showed that the baseline diaphragm thickness in the interventional group was (1.98 ± 0.52) mm and after 48 hours of phrenic nerve stimulation increased to (2.20 ± 0.45) mm (p=0.001). The baseline diaphragm thickness of (2.00 ± 0.33) mm decreased in the control group after 48 hours of mechanical ventilation to (1.72 ± 0.20) mm (p<0.001).

**Conclusions:**

Our study demonstrates that induced contraction of the diaphragm by pacing the phrenic nerve not only reduces the rate of its atrophy during mechanical ventilation but also leads to an increase in its thickness – the main determinant of the muscle strength required for spontaneous ventilation and successful ventilator weaning.

*Trial registration:* The study was registered with ClinicalTrials.gov (18/06/2018, NCT03559933, https://clinicaltrials.gov/ct2/show/NCT03559933).

**Supplementary Information:**

The online version contains supplementary material available at 10.1186/s12890-021-01677-2.

## Background

Mechanical ventilation (MV) is one of the most common forms of organ support routinely administered in the intensive care unit (ICU), with the proportion of patients requiring MV reaching up to 40% [1–3]. Mechanical ventilation has a number of adverse effects such as ventilator-associated pneumonia [4], lung injuries [5, 6], and a recently widely studied issue known as ventilator-induced diaphragmatic dysfunction (VIDD) [7, 8]. The time required to wean patients from MV is directly proportional to ICU length of stay (LOS) which increases morbidity, mortality, and healthcare costs [5, 6, 9]. Almost half of ventilated patients have difficult or prolonged weaning [10]. General muscle weakness is a common problem in patients hospitalized in the ICU [11, 12]. While muscle wasting in the limbs is a rather gradual process which typically develops over weeks [13], diaphragmatic atrophy and dysfunction appear more rapidly [14, 15]. VIDD is thought to be a complex process caused not only by muscle inactivity during MV, but also associated with many other risk factors such as malnutrition [16], sepsis or other systemic infections [17], and a number of intravenous drugs commonly used in intensive care such as neuromuscular blockers [18] and glucocorticoids [19]. Diaphragmatic muscle thinning is an essential component of VIDD [20, 21]. Among the methods for assessing the thickness of the diaphragm and its excursion during the respiratory cycle, ultrasonography has proved to be the most effective. It is a non-invasive, easily accessible examination with the possibility of repeated measurements [22].

Recently, various possibilities for the prevention or treatment of diaphragm atrophy have been explored. Stimulation of the phrenic nerves leading to contractions of the diaphragm seems to be a promising approach. Surgically implanted diaphragmatic stimulation systems have long been known and are used in some specific neurological diseases [23], but they are not suitable for temporary therapy. In the field of temporary, minimally invasive phrenic nerve stimulation, a transvenous approach using a special central venous catheter inserted into the left subclavian vein seems very promising [24, 25]. Another possibility currently being explored is a use of transcutaneous magnetic stimulation [26]. Bilateral phrenic nerve stimulation is achieved using the repetitive cervical magnetic stimulation approach on the neck. Although this would be a suitable solution because magnetic stimulation is not painful, this approach has so far only been studied in healthy, spontaneously ventilating volunteers and its feasibility among critically ill patients on MV in unknown.

Stimdia Medical, Inc., has developed the novel Percutaneous Electrical Phrenic Nerve Stimulation (PEPNS) System, which uses transcutaneous lead insertion close to the phrenic nerve in the neck region using ultrasound navigation and its feasibility and effectiveness has been demonstrated in a human, multicenter, non-randomized study [27]. The clinical study protocol evaluated the effect of stimulation on diaphragm thickness using a standardized technique consisting of repeated measurements of its thickness by ultrasound, in the eighth or ninth intercostal space, each time in the same place on the diaphragm [28–30]. Due to the routine use of ultrasound measurement of the diaphragm and its excursion as a predictor of successful weaning and extubation in our clinic, and in order to increase the robustness of stimulated patients' data, we also measured a control group of patients with comparable demographic data who did not receive diaphragmatic stimulation.

The aim of the study was to determine the effectiveness of diaphragm stimulation in increasing its thickness as a potential prevention of diaphragm atrophy in patients on mechanical ventilation with respiratory failure.

## Methods

### Design and setting

The prospective, interventional, controlled, double-center study was conducted at the Department of Anesthesiology and Intensive Care, 1st Faculty of Medicine of Charles University and Military University Hospital in Prague, Czech Republic, and at the Department of Anaesthesia and Critical Care, Royal College of Surgeons in Ireland, Beaumont Hospital, Dublin, Ireland. The study was approved by Institutional Review Boards at both institutions (Study Protocol in detail in Additional file 1).

### Participants and interventions

The interventional group (N=12) consisted of two unilaterally and ten bilaterally stimulated patients, four were predominantly ventilated on spontaneous modes such as pressure support ventilation (PSV), while the remaining eight patients were on assist-control ventilation (ACV) or some combination of ACV and PSV during stimulation days. Using ultrasound guidance multipolar stimulation electrodes were inserted near the phrenic nerve in the neck area. Due to the long time required to obtain informed consent from patients' relatives, at study enrollment (hour 0) – patients had spent an average of 165 hours on mechanical ventilation. The intervention group received stimulation therapy using the PEPNS system [27], with six, two-hour stimulation treatment sessions occurring over 48 hours. During treatment every fourth breath was stimulated and the stimulation current was adjusted to keep the patients' work of breathing within 0.2-2 joules/L. The PEPNS system recognized the onset of inspiration, regardless of the ventilation mode, using the system's airway flow sensor which triggered bilateral stimulation of the phrenic nerves. Stimulation ceased when the patient started to exhale as determined again by the flow sensor. The effectiveness of stimulation was continuously monitored by changes in tidal volume and increase in WOB. The majority of patients had trauma as the leading diagnosis (nine out of twelve) seven of whom had traumatic brain injury (TBI), the remaining patients had sepsis, rupture of the arteriovenous malformation, and lung infection (details in Additional file 2).

In the control group (N=10) of non-stimulated patients, nine were on ACV during the enrollment period, and one was exclusively on PSV mode. Patients in the control group who met inclusion criteria were consecutively enrolled in the study during a similar time window as the intervention group. The average time spent on mechanical ventilation before enrollment in the study was 159 hours. As with the stimulated group, the majority of patients had traumatic and/or neurosurgical diagnoses (seven out of ten). The others were after extensive, complicated abdominal surgery, and one patient was treated for respiratory infection (details in Additional file 2). The comparison of demographic characteristics of the interventional and control group is presented in Table 1.

**Table 1:** The demographic characteristics of the interventional and control study groups

Parameter	Intervention group	Control group	*p*
N	12	10	—
Sex (Male:Female)	11:1	6: 4	—
Age (years)	61.9 ± 7.5	60.2 ± 9.9	0.65
Weight (kg)	89.3 ± 24.4	82.5 ± 12.8	0.43
Height (cm)	174.7 ± 6.7	173.9 ± 7.3	0.80
BMI (kg·m^–2^)	29.1 ± 6.6	27.3 ± 3.8	0.46
Time on ventilator before the study (hours)	165 ± 67	159 ± 37	0.82

The values are presented as mean ± standard deviation

N—number of subjects; BMI—Body Mass Index

### Measurement

**Fig. 1:**
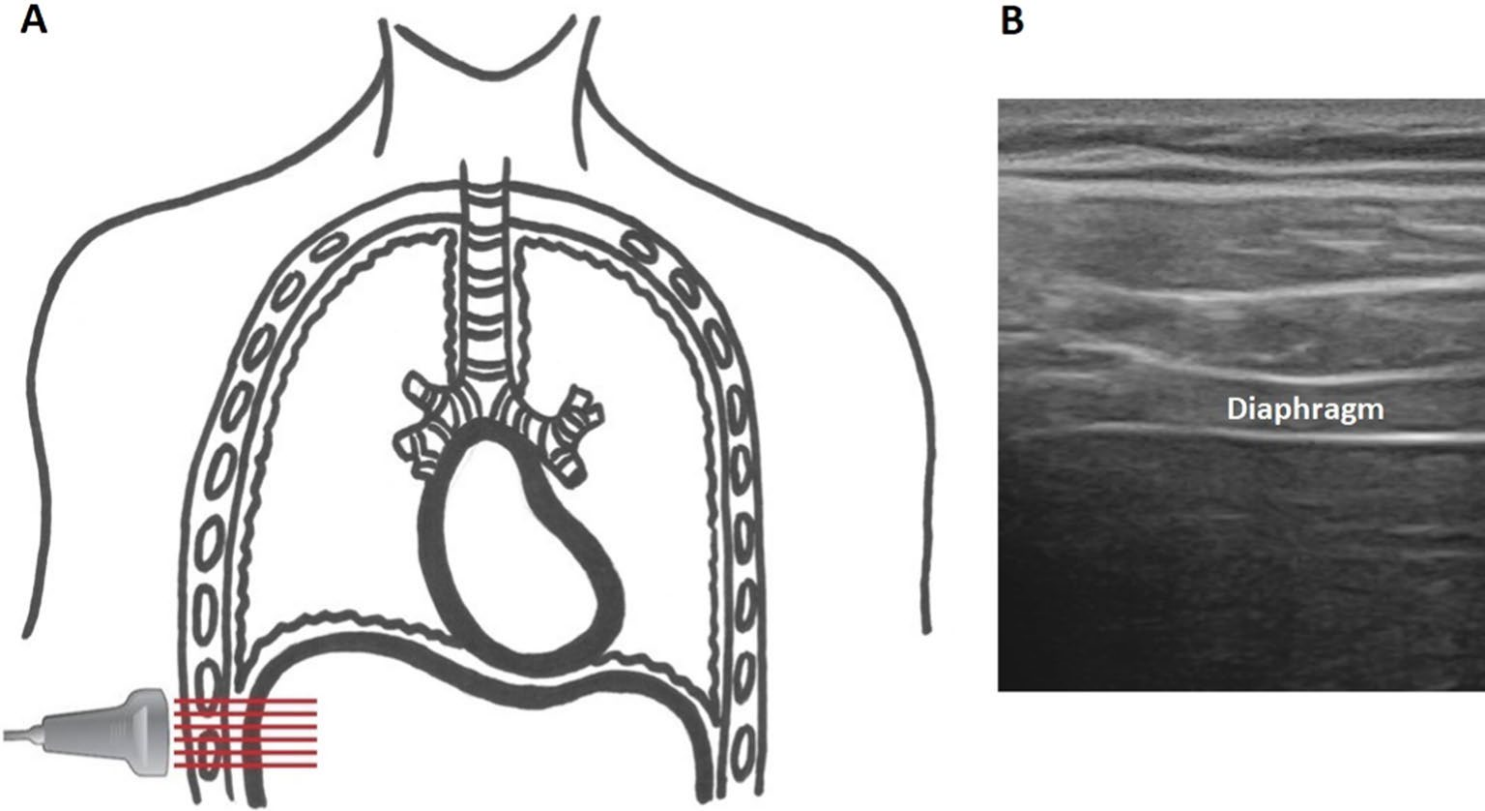
Ultrasound linear probe orientation **(a)** and view of the diaphragm **(b)** identified as a 3-layer structure comprised of two hyperechoic lines representing the pleural and peritoneal membranes and a middle hypoechoic layer representing the diaphragm muscle itself

Diaphragm thickness was measured using a standardized ultrasound technique [28–30] and imaging was performed using B-mode with a linear probe at 10-15 MHz at the zone of apposition between the eighth or ninth intercostal space on the both sides in the midaxillary line (Fig. 1).

**Fig. 2:**
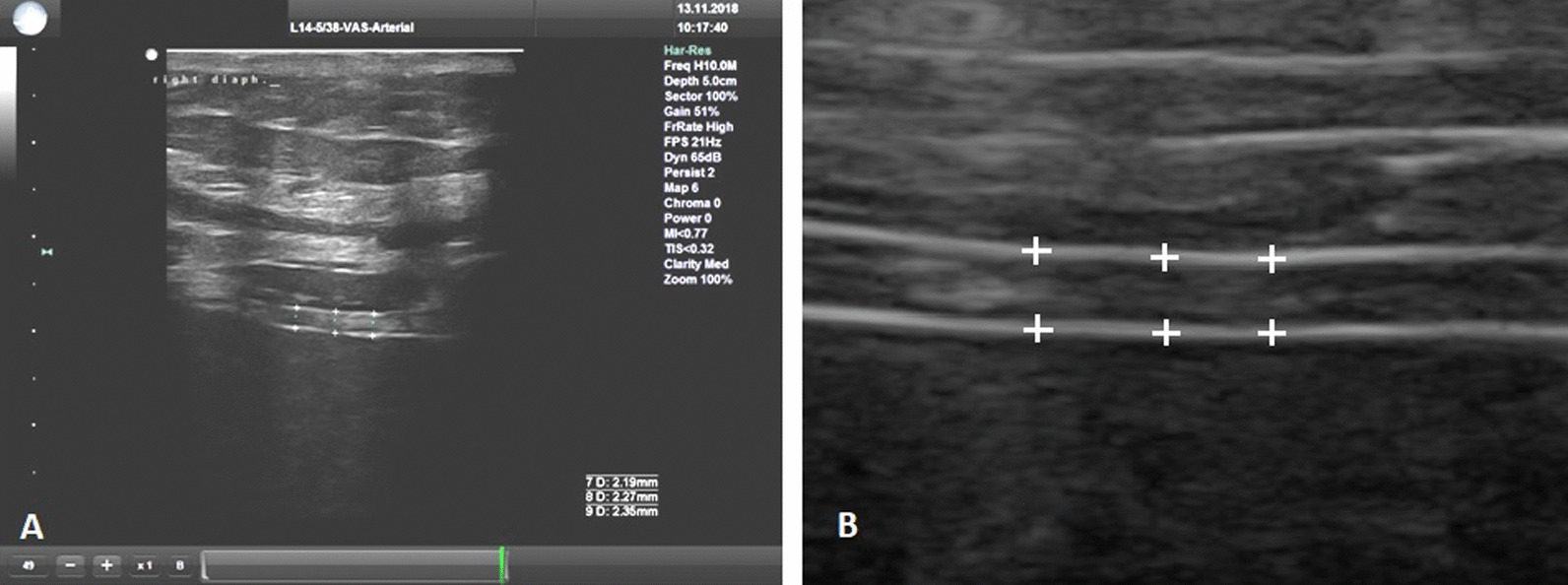
Ultrasound measurement of the right side of the diaphragm, linear probe, 10 MHz **(a)**, measurement was made from the middle of the pleural line to the middle of the peritoneal line **(b)** detail

Diaphragm thickness was measured once a day at baseline (0 hours), after 24±4 hours, and after 48±4 hours, always at the end of expiration during relaxation of the diaphragm prior to the initiation of the next breath. The measurement was performed on three separate breaths with three measurements attempted on each breath when possible. To increase accuracy, the measurements were performed in three different ways. At the end of expiration, the ultrasound image was frozen and a total of three values were measured in different parts of the diaphragm (Fig. 2). This was done over a total of three separate breath cycles. The calculated average value of diaphragm thickness was then recorded. The image documentation of the entire measurement was then saved to disk for further analysis. This consisted of a manual control measurement in the locations originally marked by the electronic caliper of the ultrasound device followed by measurement of other locations along the course of the diaphragm using the acquired images from the scan. These measurements were performed by a different physician than the one who performed the original ultrasound. In contrast to published data from the intervention group [27], data from patients who received only left phrenic nerve stimulation were also statistically processed.

### Statistical analysis

Results are presented as mean ± standard deviation (SD). Normal distribution of data was confirmed using the Shapiro-Wilk test (Statistica v7.1, StatSoft, Inc., Tulsa, OK, USA). The statistical significance of the diaphragm thickness in time within the study groups was tested using ANOVA for repeated measurements followed by Fisher’s least significant difference post-hoc tests (Statistica v7.1). A paired T-test was used for testing the statistical significance of overall diaphragm thickness before and after the experiment. Statistical differences with p values less than 0.05 by two-tailed tests were considered significant.

## Results

Diaphragm thickness was analyzed in terms of % change in thickness from baseline (0 hours), then at 24 ± 4 hours, and at 48 ± 4 hours. Using this approach allowed comparison of the effect of electrical stimulation between patients by minimizing the influence of the natural variability in diaphragm thickness between patients (diaphragm thicknesses from all measurement are in detail in Additional file 3).

From the interventional group, patient 05 was measured only on the left side since the right side was not stimulated after early lead removal. Patient 06 was excluded from the analysis due to difficulty in acquiring a quality ultrasound image due to extensive pleural effusion and body habitus.

During the experiment, the original diaphragm thickness (i.e. the baseline) in the interventional group was (1.98 ± 0.52) mm and after 48 hours of phrenic nerve stimulation increased to (2.20 ± 0.45) mm (p=0.001). In the control group the original diaphragm thickness of (2.00 ± 0.33) mm decreased after 48 hours of mechanical ventilation to (1.72 ± 0.20) mm (p<0.001).

**Fig. 3:**
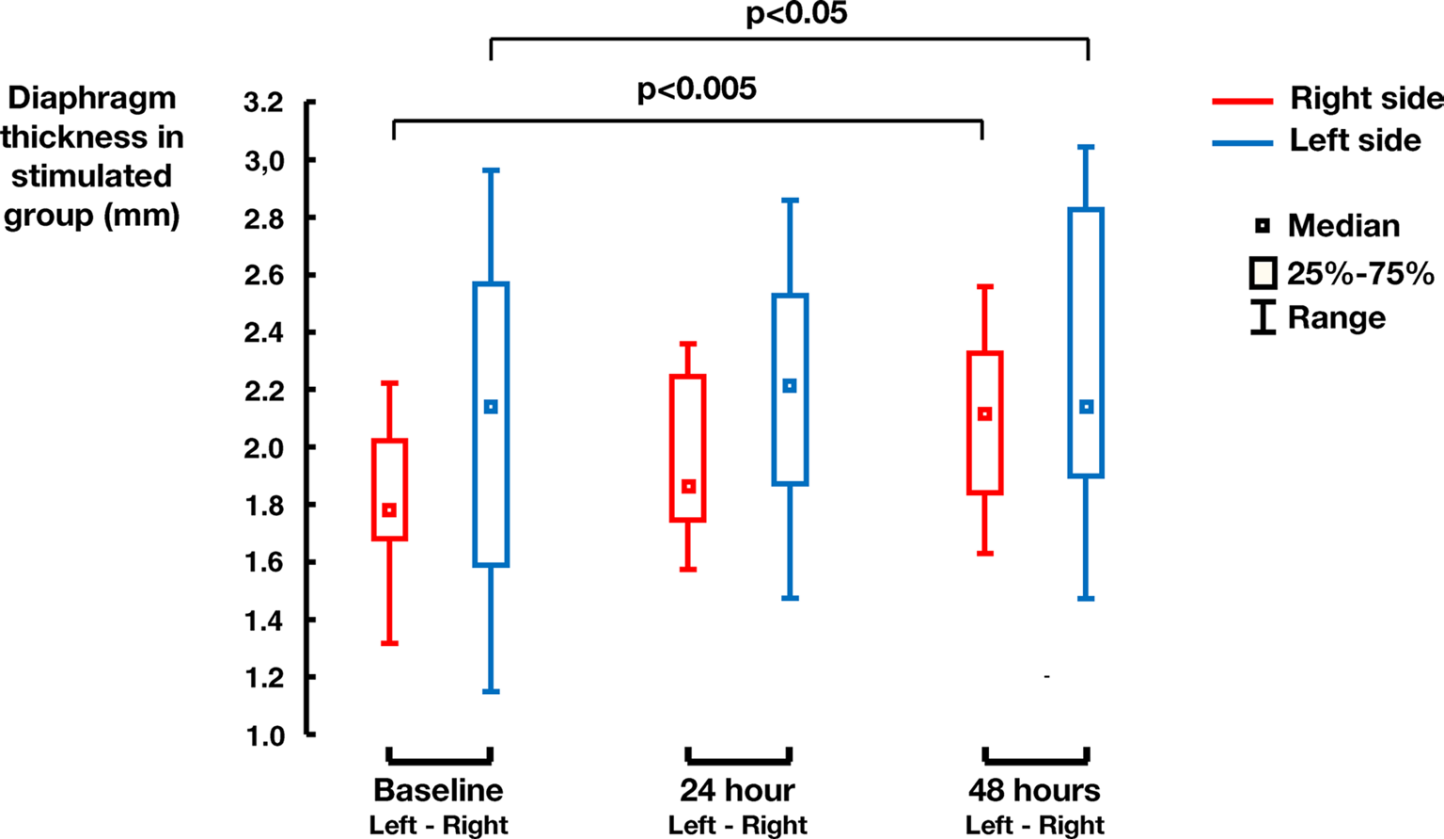
Increase in diaphragm thickness in the interventional group

**Fig. 4:**
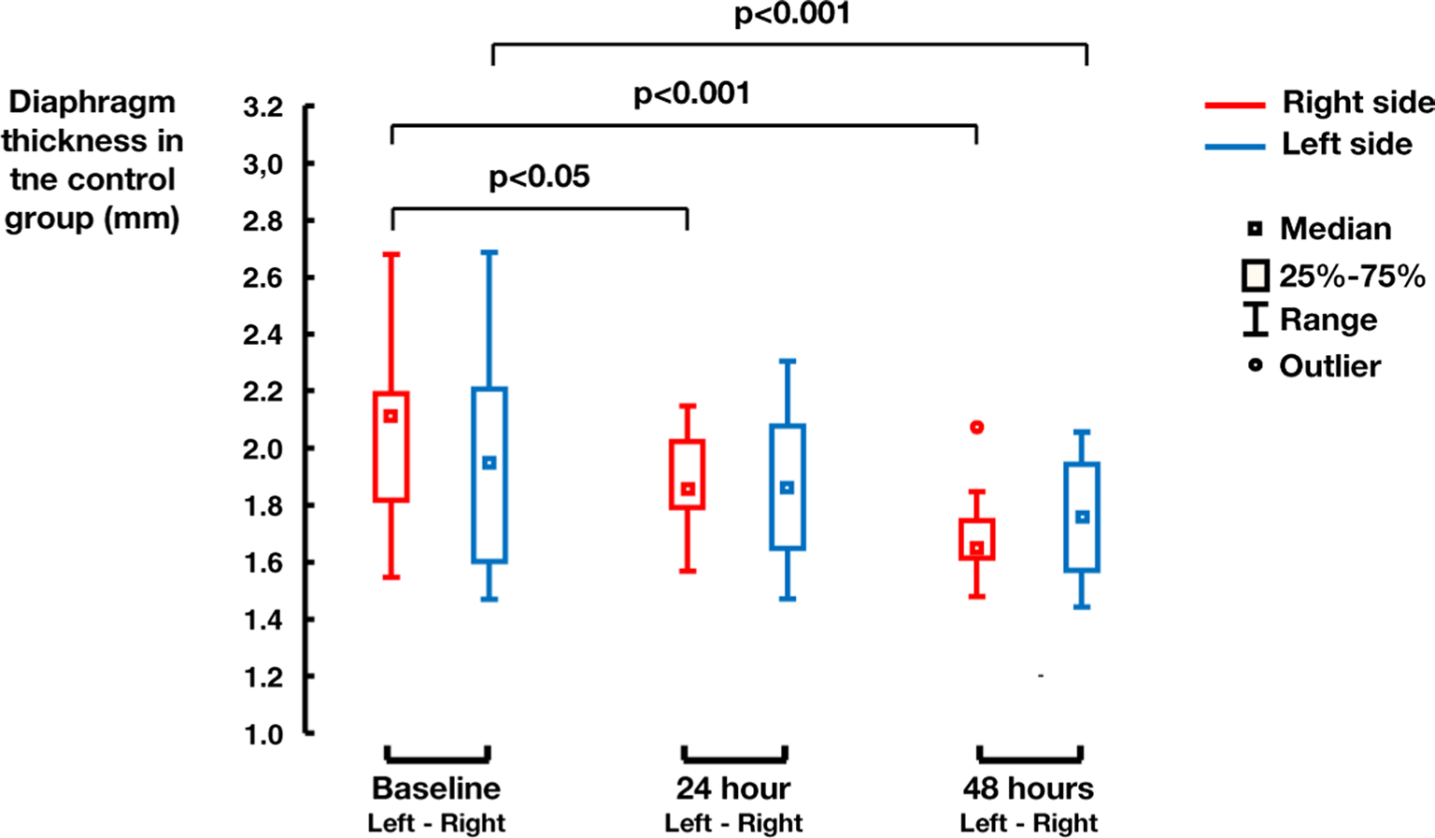
Decrease in diaphragm thickness in the control group

The details of changes in diaphragm thickness during the experiment are presented in Fig. 3 for the interventional group and in Fig. 4 for the control group. The results are presented separately for right and left side measurements.

**Fig. 5:**
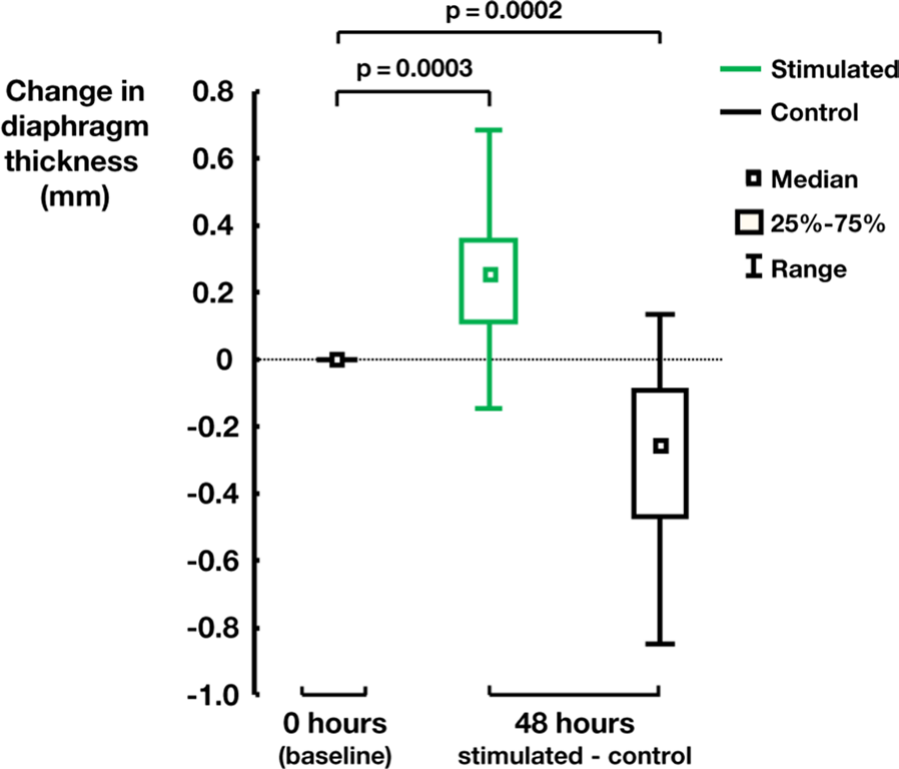
Increase in diaphragm thickness after 48 hours with stimulation and its corresponding decrease after 48 hours in the control group (without stimulation) compared to baseline at hour 0

Figure 5 shows the overall increase (right and left together) in diaphragm thickness in the interventional group after 48 hours. This increase was statistically significant (p=0.0003). By 48 hours, the diaphragm thickness was on average almost 15% thicker than at baseline in the interventional group and 12% thinner than at baseline in the control group (p = 0.0002).

## Discussion

The principle finding in this study was that ICU patients who received phrenic nerve stimulation during mechanical ventilation experienced an increase in diaphragm muscle thickness, whereas similar ventilated patients who did not receive stimulation experienced a corresponding decrease in thickness. This effect was observed on both sides of the diaphragm with a nearly 15% increase in diaphragm thickness seen in the stimulated group and a more than 12% decrease in thickness seen in the control group.

The thickness of the diaphragm progressively decreases during assist-control ventilation (ACV) modes and, conversely, increases during pressure support ventilation (PSV), when all breaths are initiated by the patient's spontaneous respiratory activity [30]. Francis et al. observed a reduction in diaphragm thickness in patients on ACV of approximately 5-6% per day, which is consistent with previous findings by Grosu et al. [31] and a gradual increase among patients who started on ACV during the study and continued on PSV. Another interesting variable is the level of PEEP used. It has been hypothesized that lung volume at the end of expiration with the use of PEEP puts the passive diaphragm in a contracted position, so it is possible that the diaphragm atrophied at a relatively faster rate than in patients not on PEEP [30]. However, because the use of PEEP for prevention of lung atelectasis [32] is standard practice in the ICU, it is not possible to investigate its effect on the rate of diaphragm atrophy. In our study, it should be noted that in both groups the patients were predominantly ventilated on ACV mode, but some were irregularly switched to PSV during the day/night so it is not possible to precisely quantify the effect of ventilation mode on the change in diaphragm thickness. In addition, some patients on PSV in the control group in contrast to the findings of previous studies still experienced a reduction in diaphragm thickness. In addition, one patient from the intervention group had been on PSV for many days prior to enrollment in the study, but nevertheless responded to 48 hours of stimulation with a significant increase in diaphragm thickness. Thus there is likely an effect not only of the selected ventilation mode (ACV or PSV), but also of the intensity of ventilation support itself as well as the selected PEEP level. In addition, the authors believe that “external” stimulation of the phrenic nerve allows the involvement of much larger muscular units of the diaphragm leading to more effective contraction than when the stimulus is induced spontaneously (i.e. physiologically from the brain center). This assumption allows for the possibility of the use of phrenic nerve stimulation for active rehabilitation of the diaphragm even in patients who are exclusively on PSV, i.e. those who are already in the advanced phase of weaning. The possibility of using low stimulation currents allows for tolerance of stimulation even in fully conscious patients. However, precise insertion of the stimulation electrodes in the immediate vicinity of the phrenic nerve is required to achieve this.

We performed multiple measurements of diaphragm thickness, first using the cursor of the ultrasound device, and later manually on the acquired images including measurements in additional parts of the diaphragm which were verified by an independent physician. The reason was to exclude subjective variability in measurement and relative error of the ultrasound device’s measurements which were, in any case, expected to be very low (+/- 0.36 %) using a linear probe with a frequency of 10 Hz, and measuring in the axillary plane [33].

The study has several limitations. First, although the increase in diaphragm thickness in the stimulated group was significant, as was the decrease in the control group, but because of the relatively large variability in diaphragm thickness in patients on mechanical ventilation described in the literature [34], it would be appropriate to verify this in a larger sample of patients. Unfortunately, the sample size was limited by the number of patients enrolled in the intervention group. Second, the stimulation protocol was designed to pace the diaphragm every fourth breath in two-hour sessions spaced every eight hours for 48 hours. It would be appropriate to try other timing protocols and to estimate how long of a stimulation period would be suitable for a particular patient. It is very likely that even with the current stimulation schedule, most patients would require much longer stimulation times to achieve clinically significant results. It would certainly be interesting to compare individual subgroups of stimulated patients with each other, according to the presence or absence of a primary pulmonary pathology. It can be assumed that patients with lung disease could benefit from stimulation more than those without it. Unfortunately, our group of patients is too small to draw these conclusions. Third, almost all patients in the intervention group were sedated during stimulation sessions. Although patients were regularly scored for possible signs of pain using the CPOT (Critical Care Pain Observation Tool) scoring system and no signs of pain were recorded during stimulation, only one patient was fully conscious. Therefore, to find a safe painless stimulation current limit, it would be necessary to enroll more unsedated patients. Fourth, because ventilation modalities have not been standardized and cannot be determined the proportion of spontaneous effort and the level of inspiratory effort, they may have differed between groups. However, given that routine intensive care of the ventilated patient proceeded in the same way, i.e. there were changes in ventilatory support due to the intermittent need to deepen sedation for intensive care procedures (patient transport, invasive interventions, hygiene procedures, etc.), we believe that there were no significant differences between the intervention and control groups.

Our previous experience with diaphragm stimulation led us to the idea of ​​using this method in patients with respiratory failure presenting a background of chronic lung disease such as COPD. Exacerbation of this disease due to infection usually needs targeted antibiotic or antifungal therapy, which requires at least several days to achieve clinical effect. Unfortunately, this time may necessarily be spent on MV leading to an acceleration of diaphragmatic atrophy and therefore may prolong or completely prevent successful ventilator weaning. Hospital mortality in mechanically ventilated patients with COPD is almost 25%, 1-year mortality approaches 40%, and 5-year mortality exceeds 70% [35]. These patients often eventually require a tracheostomy and long-term intensive care dependent on ventilatory support for weeks or more. If such patients were stimulated at the initiation of MV, we could gain time for pharmacological therapy of the infection to take effect without an increased risk of prolonged weaning due to diaphragm atrophy and long-term MV with all of its possible consequences. Unfortunately, due to the lengthy process in obtaining informed consent from the patients' relatives, we were able to start stimulation in our patients after about a week on mechanical ventilation. The authors believe that there are a number of other diseases where stimulation of the diaphragm in sedated patients on MV would be appropriate. The aim of further research should therefore be to look for indications for individual diseases and find out which patients could benefit the most from this method.

## Conclusion

Our study demonstrates that induced contraction of the diaphragm by pacing the phrenic nerve has potenital not only to reduce the rate of its atrophy during mechanical ventilation but, in fact, probably leads to an increase in its thickness — the main determinant of the muscle strength required for spontaneous ventilation. Percutaneous electrical phrenic nerve stimulation represents a promising new approach to maintaining diaphragm strength and may offer a future option for preventing or even treating ventilator induced diaphragm dysfunction in patients on mechanical ventilation. However, we recognize that this parameter is only one piece of a larger puzzle in the weaning process and does not necessarily lead to a reduction in time on the ventilator. Our findings, nevertheless, open the way for further research in this field.

## Supplementary Information

**Additional file 1.** Study Protocol.

**Additional file 2. **Detailed patient demographics.

**Additional file 3.** Diaphragm Thickness Measurements.

## Data Availability

All data generated or analyzed during this study are included in this publisher article in its supplementary information files.

## Abbreviations

ACV: Assist-control ventilation
BMI: Body mass index
CPOT: Critical care pain observation tool
COPD: Chronic obstructive pulmonary disease
ICU: Intensive care unit
MV: Mechanical ventilation
N: Number of subjects
LOS: Length of stay
PEEP: Positive end expiratory pressure
PEPNS: Percutaneous electrical phrenic nerve stimulation
PSV: Pressure support ventilation
SD: Standard deviation
TBI: Traumatic brain injury
VIDD: Ventilator-induced diaphragmatic dysfunction
