# Supplementary material for: Phrenic nerve stimulation prevents diaphragm atrophy in patients with respiratory failure on mechanical ventilation
Source: BMC Pulm Med. 2021 Oct 8;21:314. doi: 10.1186/s12890-021-01677-2 (PMC8500254; doi:10.1186/s12890-021-01677-2)
Supplement: Supplementary file 1 — Additional file 1. Study Protocol. [file 12890_2021_1677_MOESM1_ESM.docx]

***Additional file 1***

**Study PROTOCOL**

**Part A - The interventional group**

Summary of *Percutaneous Electrical Phrenic Nerve Stimulation (PEPNS) System Feasibility Study* Protocol

*Study Sponsor:*

- Stimdia Medical, Inc.

*Study Device:*

- Percutaneous Electrical Phrenic Nerve Stimulation (PEPNS) System.

*Rationale:*

- The diaphragm atrophy and dysfunction is a major problem among critically ill patients on mechanical ventilation. Ventilator induced diaphragmatic dysfunction is thought to play a major role in this process resulting in a failure of successful weaning. Stimulation of the phrenic nerves leading to the diaphragm contraction could prevent or treat this atrophy.

*Purpose:*

- The purpose of this study is to evaluate the safety and performance of the PEPNS System in patients that need to be mechanically ventilated for at least 48 hours and up to 7 days in the Intensive Care Unit (ICU).

*Study Design:*

- Prospective; Non-randomized; Open-label

*Enrollment:*

- Up to 2 sites and up to 10 subjects per site. The very first 2 subjects will be stimulated on the left phrenic nerve and followed for 30 days prior to enrolling additional patients. In addition, compound muscle action potential testing (CMAP) and nerve conduction studies, will be conducted on the first 2 subjects during the index procedure and at or before the 30 day follow up.

*Follow Up:*

- All subjects will be followed up for 30 days after their index procedure for a total participation period of up to 41 days.

*Study Population:*

- Patients that need to be mechanically ventilated for at least 48 hours and up to 7 days in the Intensive Care Unit (ICU).

*Inclusion Criteria:*

- 18 years or older (Adult).
- Male or Female.
- Able and willing to give informed consent or whose legally authorized representative is able and willing to give informed consent.
- Subject who in the opinion of the admitting consultant/intensivist is likely to be ventilated for > 48 hours from time of recruitment since study treatment will be for up to 48 hours.

*Exclusion Criteria:*

- Subject has a left ventricular ejection fraction (LVEF) < 20%.
- Subject unlikely to survive 72 hours due to coexisting medical conditions.
- Subject has an implanted pulse generator or implanted electronic device: Examples: Cardiac pacemaker, Defibrillator, ICD, Watchman, Vagal nerve stimulator, Spinal cord stimulator, Gastric stimulator or Diaphragmatic stimulator.
- Subject has experienced an Acute Myocardial Infarction (AMI) within 72 hours prior to this screening or patient is on high dose inotropic support or subject is deemed to be in cardiogenic shock.
- Subject has significant bleeding diathesis, or is at risk of significant haemorrhage, patient is receiving full dose systemic anticoagulation
- Subject has a known or suspected phrenic nerve paralysis or neuromuscular or inflammatory muscle diseases where the diaphragm itself may not be functional.
- Subject has an active systemic infection or local infection at or around the insertion site. Subject is neutropenic or has signs of significant immunocompromise.
- Subject is known or suspected to be pregnant or is lactating.
- Subject will be unavailable for, or is unwilling to comply with, follow up requirements of the protocol.
- Subject is currently enrolled or is expected to be enrolled in any other study of an investigational drug or device who has received treatment under that protocol with the investigational product during the 30 days prior to screening.
- Subject has undergone a surgery or interventional procedure within the neck region aside from placement of an internal jugular (IJ) vein catheter.
- Subject has been diagnosed and has been treated for neck cancer within the past 5 years.
- Subject is known to have a demonstrated intra cardiac thrombus on echocardiography.
- Subject has uncontrolled hyperthyroidism, hypertension.
- Subject has had any cerebral ischemic event (Stroke or Transient Ischemic Attack TIA) in the 6-month interval preceding the screening date.
- Subject has degenerative nerve disorders such as amyotrophic laterals sclerosis (ALS).
- Subject has an elevated hemidiaphragm on chest x-ray.
- Subject written informed consent not obtained.

*Primary Endpoints:*

- Capture of the Left and/or Right Phrenic Nerve > 80% with an output parameter of < 10.5 volts.
- WOB (Work of Breathing) kept between 0.3 and 0.7 joules/L for 80% of breaths.

*Secondary Endpoints:*

- The percentage of patients who receive safe and successful placement of the multipolar lead in the left and right phrenic nerve utilizing ultrasound guidance will be determined.
- Phrenic nerve stimulation in synchrony with Mechanical Ventilation (MV) breaths will be measured to verify that is occurs with inspiration.
- The percentage of patients who experience one or more serious device/procedure-related adverse events during the study will be reported.

*Additional Study Data:*

- Time to weaning from mechanical ventilation data will be collected.
- Diaphragm thickness at 0, 24 and 48 hours.

**Part B - The control group**

*Purpose:*

- The purpose of this study is to evaluate the diaphragm thickness in patients who did not receive diaphragmatic stimulation.

*Study Population:*

- Patients that need to be mechanically ventilated for at least 48 hours and up to 7 days in the Intensive Care Unit (ICU).

*Enrollment:*

- One site, number of subject the same as bilaterally stimulated patiens (up to 20).

*Inclusion Criteria:*

- Inclusion criteria identical to part of protocol A.
- Due to the routine use of ultrasound measurement of the diaphragm and its excursion as a predictor of successful weaning and extubation in our clinic, informed consent is not needed.

*Exclusion Criteria:*

- Exclusion criteria identical to part of protocol A.

*Primary Endpoint:*

- Change in diaphragm thickness at 0, 24 and 48 hours.
