# Supplementary material for: Phrenic nerve stimulation prevents diaphragm atrophy in patients with respiratory failure on mechanical ventilation
Source: BMC Pulm Med. 2021 Oct 8;21:314. doi: 10.1186/s12890-021-01677-2 (PMC8500254; doi:10.1186/s12890-021-01677-2)
Supplement: Supplementary file 2 — Additional file 2. Detailed patient demographics. [file 12890_2021_1677_MOESM2_ESM.docx]

***Additional file 2***

***Supplementary Table 1:*** *Detailed patient demographics*

| **Patient ID** | **Mode of Ventilation** | **Time on vent before Stimulation (hours)** | **Sex** | **Age** | **Weight (kg)** | **Height (cm)** | **BMI** | **Diagnosis** |
| --- | --- | --- | --- | --- | --- | --- | --- | --- |
|  |  |  |  |  |  |  |  |  |
| P01S02 | PSV/CPAP | 89 | MALE | 70 | 60 | 168 | 21.3 | TBI, COPD, Chronic alcohol abuse |
| P02S02 | PSV | 163 | MALE | 56 | 100 | 171 | 34.2 | Fall, Evacuation of subdural hematoma |
| P03S01 | BIPAP, SIMV | 139 | MALE | 74 | 85 | 180 | 26.2 | Rupture of AV malformation |
| P04S01 | BIPAP, SIMV, PSV | 132 | MALE | 58 | 90 | 175 | 29.4 | Pneumonia, Trauma, TBI |
| P05S02 | PRVC-SIMV,PSV | 144 | MALE | 64 | 127 | 185 | 37.1 | TBI |
| P06S02 | PSV | 218 | MALE | 64 | 97 | 180 | 29.9 | Trauma, postoperative |
| P07S02 | SIMV, PRVC | 173 | MALE | 51 | 65 | 173 | 21.7 | TBI, Pneumonia |
| P08201 | SIMV, PSV | 142 | MALE | 61 | 90 | 180 | 28.0 | Trauma, TBI |
| P09202 | PSV | 269 | MALE | 56 | 140 | 178 | 44.2 | COPD, ARF, CRF, sepsis |
| P10S02 | SIMV, PSV | 101 | MALE | 56 | 70 | 171 | 23.9 | Trauma |
| P11S01 | SIMV, PSV | 103 | MALE | 59 | 80 | 175 | 26.1 | TBI, Pneumonia, COPD |
| P12201 | PSV | 307 | FEMALE | 74 | 68 | 160 | 26.6 | COPD, ARF, Pneumonia, aspiration |
| P01C | SIMV | 112 | MALE | 62 | 82 | 168 | 29.1 | TBI |
| P02C | SIMV, PSV | 96 | MALE | 55 | 91 | 182 | 27.5 | TBI |
| P03C | SIMV | 156 | FEMALE | 58 | 100 | 173 | 33.4 | Multiple tauma, TBI |
| P04C | PRVC-SIMV | 188 | FEMALE | 77 | 65 | 164 | 24.2 | Abdominal surgery, sepsis |
| P05C | SIMV, PSV | 123 | MALE | 45 | 73 | 174 | 24.1 | COPD, pneumonia |
| P06C | SIMV, PSV | 172 | MALE | 62 | 85 | 178 | 26.8 | Subarachnoideal haemorhage |
| P07C | PSV | 202 | MALE | 67 | 94 | 185 | 27.5 | TBI |
| P08C | SIMV | 166 | FEMALE | 72 | 86 | 164 | 32.0 | TBI |
| P09C | SIMV, PRVC, PSV | 179 | FEMALE | 49 | 60 | 171 | 20.5 | Abdominal surgery, sepsis |
| P10C | SIMV, PSV | 201 | MALE | 55 | 89 | 180 | 27.5 | TBI, trauma |

Abbreviations: P01–P10—patient 01–10, S01—site 01 (Military University Hospital Prague, Czech Republic), S02—site 02 (Beaumont Hospital, Dublin, Ireland), P01C–P10C—patient 01–10 control group, COPD—chronic obstructive pulmonary disease, TBI—trauma brain injury, ARF/CRF—acute/chronic renal failure.
