# Supplementary material for: Phrenic nerve stimulation prevents diaphragm atrophy in patients with respiratory failure on mechanical ventilation
Source: BMC Pulm Med. 2021 Oct 8;21:314. doi: 10.1186/s12890-021-01677-2 (PMC8500254; doi:10.1186/s12890-021-01677-2)
Supplement: Supplementary file 3 — Additional file 3. Diaphragm Thickness Measurements. [file 12890_2021_1677_MOESM3_ESM.docx]

***Additional file 3 Supplementary Table 2:*** *Diaphragm Thickness Measurements*

| Patient ID | Side | Average Thickness (mm) | | | Fractional change | |
| --- | --- | --- | --- | --- | --- | --- |
|  |  | **Baseline** | **24 Hours** | **48 Hours** | **0-24 Hours** | **0-48 Hours** |
|  |  |  |  |  |  |  |
| P01S02 | L | 2.354 | 2.232 | 2.141 | -0.0518 | -0.0905 |
|  | R | 2.106 | 2.140 | 2.247 | +0.0161 | +0.0670 |
| P02S02 | L | 2.556 | 2.769 | 2.576 | +0.0833 | +0.0080 |
|  | R | 2.561 | 2.624 | 2.799 | +0.0246 | +0.0929 |
| P03S01 | L | 2.571 | 2.429 | 2.928 | -0.0552 | +0.1386 |
|  | R | 1.738 | 1.820 | 1.901 | +0.0472 | +0.0938 |
| P04S01 | L | 2.143 | 2.044 | 2.100 | -0.0462 | -0.0201 |
|  | R | 2.181 | 2.150 | 2.241 | -0.0142 | +0.0275 |
| P05S02 | L | 2.925 | 2.859 | 3.045 | -0.0226 | +0.1200 |
|  | R | 2.701 | 2.702 | 2.647 | +0.0004 | -0.0200 |
| P07S02 | L | 1.422 | 1.692 | 1.686 | +0.1899 | +0.8338 |
|  | R | 1.620 | 1.575 | 2.011 | -0.0278 | +0.2414 |
| P08S01 | L | 1.748 | 1.869 | 1.896 | +0.0692 | +0.0847 |
|  | R | 1.769 | 1.730 | 2.224 | -0.0220 | +0.2572 |
| P09S02 | L | 2.963 | 2.531 | 2.829 | -0.1458 | -0.0452 |
|  | R | 1.870 | 2.347 | 2.559 | +0.2551 | +0.3684 |
| P10S02 | L | 1.150 | 1.474 | 1.472 | +0.2817 | +0.2800 |
|  | R | 1.318 | 1.756 | 1.630 | +0.3323 | +0,2367 |
| P11201 | L | 1.723 | 2.214 | 2.342 | +0.2850 | +0.3593 |
|  | R | 2.222 | 2.360 | 2.418 | +0.0621 | +0.0882 |
| P12S01 | L | 1.587 | 1.874 | 1.952 | +0.1808 | +0.2300 |
|  | R | 1.787 | 1.908 | 1.776 | +0.0677 | -0.0062 |
| P01C | L | 1.944 | 1.677 | 1.437 | -0.2670 | -0.5070 |
|  | R | 2.068 | 1.815 | 1.473 | -0.1223 | -0.2877 |
| P02C | L | 1.873 | 1.982 | 2.019 | +0.5820 | +0.0780 |
|  | R | 2.139 | 1.969 | 1.741 | -0.0795 | -0.1861 |
| P03C | L | 1.945 | 1.839 | 1.684 | -0.0545 | -0.1342 |
|  | R | 2.148 | 1.864 | 1.680 | -0.1322 | -0.2179 |
| P04C | L | 2.200 | 2.070 | 2.048 | -0.0591 | -0.6910 |
|  | R | 2.230 | 2.138 | 2.064 | -0.0413 | -0.0744 |
| P05C | L | 1.463 | 1.466 | 1.522 | +0.0021 | +0.0403 |
|  | R | 1.541 | 1.562 | 1.608 | +0.0136 | +0.0408 |
| P06C | L | 1.940 | 1.866 | 1.816 | -0.0381 | -0.0640 |
|  | R | 1.785 | 1.711 | 1.605 | -0.0415 | -0.1008 |
| P07C | L | 2.675 | 2.083 | 1.917 | -0.2213 | -0.2834 |
|  | R | 2.669 | 2.103 | 1.839 | -0.2121 | -0.3110 |
| P08C | L | 2.280 | 2.296 | 1.939 | +0.0070 | -0.1496 |
|  | R | 2.183 | 2.019 | 1.730 | -0.0751 | -0.2075 |
| P09C | L | 1.594 | 1.587 | 1.562 | -0.0044 | -0.0201 |
|  | R | 1.808 | 1.783 | 1.493 | -0.0138 | -0.3150 |
| P10C | L | 1.592 | 1.640 | 1.593 | +0.0302 | +0.0006 |
|  | R | 1.834 | 1.836 | 1.605 | +0.0011 | -0.1249 |

Abbreviations: P01–P10—patient 01–10, S01—site 01 (Military University Hospital Prague, Czech Republic), S02—site 02 (Beaumont Hospital, Dublin, Ireland), P01C–P10C—patient 01–10 control group.
